# Deep Transfer Learning Enables Robust Prediction of Antimicrobial Resistance for Novel Antibiotics

**DOI:** 10.3390/antibiotics11111611

**Published:** 2022-11-12

**Authors:** Yunxiao Ren, Trinad Chakraborty, Swapnil Doijad, Linda Falgenhauer, Jane Falgenhauer, Alexander Goesmann, Oliver Schwengers, Dominik Heider

**Affiliations:** 1 Department of Data Science in Biomedicine, Faculty of Mathematics and Computer Science, Philipps-University of Marburg, 35032 Marburg, Germany; 2 Center for Synthetic Microbiology (SYNMIKRO), Philipps-University of Marburg, 35032 Marburg, Germany; 3 Institute of Medical Microbiology, Justus Liebig University Giessen, 35392 Giessen, Germany; 4 German Center for Infection Research, Partner Site Giessen-Marburg-Langen, 35392 Giessen, Germany; 5 Institute of Hygiene and Environmental Medicine, Justus Liebig University Giessen, 35392 Giessen, Germany; 6 Hessisches Universitäres Kompetenzzentrum Krankenhaushygiene, 35392 Giessen, Germany; 7 Department of Bioinformatics and Systems Biology, Justus Liebig University Giessen, 35392 Giessen, Germany

**Keywords:** transfer learning, antimicrobial resistance, small data with imbalanced label

## Abstract

Antimicrobial resistance (AMR) has become one of the serious global health problems, threatening the effective treatment of a growing number of infections. Machine learning and deep learning show great potential in rapid and accurate AMR predictions. However, a large number of samples for the training of these models is essential. In particular, for novel antibiotics, limited training samples and data imbalance hinder the models’ generalization performance and overall accuracy. We propose a deep transfer learning model that can improve model performance for AMR prediction on small, imbalanced datasets. As our approach relies on transfer learning and secondary mutations, it is also applicable to novel antibiotics and emerging resistances in the future and enables quick diagnostics and personalized treatments.

## 1. Introduction

Antimicrobial resistance (AMR) has become one of the serious public health problems worldwide, threatening the effective treatment of a growing number of infections [1]. There were over 700,000 deaths from drug-resistant infections in 2019, and it could rise to 10 million deaths by 2050 according to estimations from the World Health Organization (WHO) [2].

Machine learning and deep learning approaches have played significant roles in antibiotic resistance prediction in recent years [3,4,5,6]. A number of deep-learning-based models and tools for predicting AMR genes or peptides have been developed, e.g., DeepARG [7] or Deep-AmPEP [8]. These methods also promoted the discovery of new antibiotics. For example, Stokes et al. trained a deep learning model based on multiple chemical libraries [9]. They found a molecule showing bactericidal activity against a broad phylogenetic spectrum of pathogens, and thus has the potential to be the basis for a new antibiotic [9]. However, skewed distribution of the data in machine learning often obstructs the accuracy and generalization of model training [10]. In fact, many datasets about medical diagnoses, such as cancer diagnostics, are imbalanced datasets and typically have a low number of samples [10]. For training a machine learning model, a large number of samples is necessary. However, these data are typically not available for novel antibiotics.

Transfer learning (TL) has shown promising applications for such challenges in recent years [11,12,13,14,15,16,17,18]. The basic idea of transfer learning is to transfer knowledge from source domains to target domains for improving the model performance [11,15,19]. In contrast to traditional machine learning (including deep learning), having only one domain and one task, transfer learning extends the notion of domain and task, in which the domains and tasks between the training and test data can be different but related in some ways [20,21,22]. Generally, the source domain is a set of data with a large number of data samples with high-quality labels. In contrast, data in the target domain may include a limited number of samples with unbalanced labels. Thus, transfer learning is widely used to solve the issue with limited datasets for visual classification and text classification [21,23,24,25,26,27]. For example, many researchers firstly trained a convolutional neural network (CNN) model on the ImageNet dataset (pre-training) and then transferred the information from the pre-trained model into a new task (fine-tuning) to solve a wide range of computer vision problems [23,24,25]. The Word2Vec dataset is also commonly used as a pre-training dataset for text classification [28]. Gupta et al. enhanced predictive analysis on small data using a cross-property deep transfer learning model [29]. Park et al. used meta-transfer learning to explore the data heterogeneity and extremely small sample size problem based on single cell data [30]. Transfer learning is also widely used in the medical area with an imbalanced label [10,31,32,33,34]. For example, Gao et al. used deep transfer learning to reduce healthcare disparities arising from imbalanced biomedical data [35]. They first trained the model on the majority group data, then transferred the knowledge learned to each minority group to improve the model performance. Thus, our study aims to transfer the knowledge from a well-trained model to a small amount of imbalanced label data to explore whether the performance for AMR prediction can be improved.

Based on our previous work [6], our models, especially the CNN, performed well for AMR prediction based on whole genome mutations, while the performance on the data with the imbalanced label can still be improved. Therefore, in our work, we firstly constructed a basic CNN model for each antibiotic in our dataset, including ciprofloxacin (CIP), cefotaxime (CTX), ceftazidime (CTZ), and gentamicin (GEN). We then used the model for CIP, i.e., the best-performing CNN, as the pre-trained model and transferred the knowledge to improve the prediction of the other three antibiotics, i.e., CTX, CTZ, and GEN (see Study design). Our results show that transfer learning can significantly improve the prediction performance on the other antibiotics. Our work also illustrates that the pre-trained model can generalize well on unseen public datasets that are extremely imbalanced, i.e., have a low number of samples for the resistance class. We provide a deep transfer learning model that can achieve accurate and robust AMR prediction on small, imbalanced datasets. By combining secondary mutation profiles and our pre-trained network, we pave the way for other training tasks concerning AMR with small, imbalanced datasets in the future, and thus enable a quick and generic solution for novel antibiotics and AMR in the future.

## 2. Results

### 2.1. Datasets

**Figure 1:**
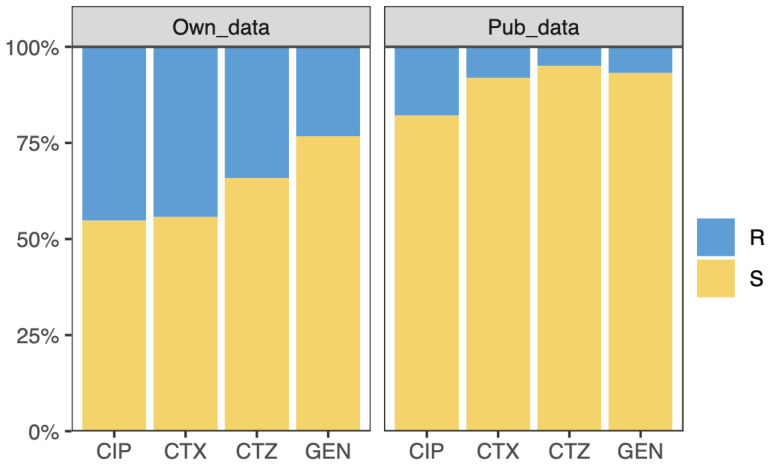
Overview of the samples. The samples are resistant (R) or susceptible (S) to ciprofloxacin (CIP), cefotaxime (CTX), ceftazidime (CTZ), and gentamicin (GEN). The left and right panel show the resistant and susceptible sample information on our and public dataset considered for this study, respectively.

In this work, we used two datasets of *Escherichia coli* (*E. coli)* with whole-genome sequencing (WGS) and resistance information for four antibiotics, namely ciprofloxacin (CIP), cefotaxime (CTX), ceftazidime (CTZ), and gentamicin (GEN). The first dataset contains 809 *E. coli* strains, produced by our laboratory. The isolates were collected from human and animal clinical samples. Antimicrobial susceptibility testing was performed using the VITEK^®^ 2 system (bioMérieux, Nürtingen, Germany) and interpreted following EUCAST guidelines. DNA isolation and whole-genome sequencing were performed as described in Falgenhauer et al. [36]. The percentage of isolates resistant to CIP, CTX, CTZ, and GEN are 45%, 44%, 34%, and 23%, respectively (see Figure 1). This dataset was split into the training dataset and testing dataset (see Section 2.2). The second dataset comprises 1509 *E. coli* strains collected from public datasets [37]. This dataset is highly imbalanced concerning resistant and sensitive isolates. The isolates that are resistant to CIP, CTX, CTZ, and GEN are 18%, 8%, 5%, and 7% of all isolates, respectively (see Figure 1). We used this dataset as the external validation dataset to demonstrate the application of transfer learning on an imbalanced, small, and unseen dataset.

### 2.2. Study Design

**Figure 2:**
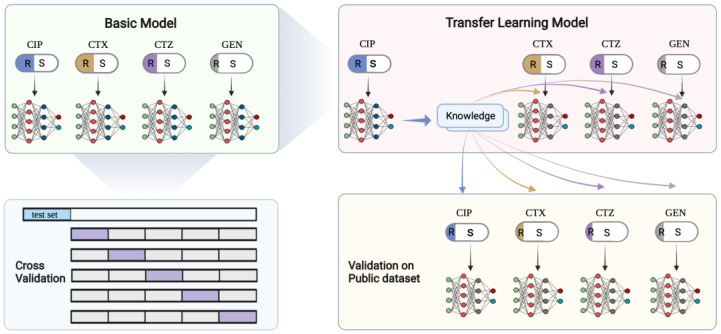
Deep transfer learning schemes. In the top left panel, the basic CNN models are shown. Each model is trained on independent antibiotics and evaluated on a new dataset. The top right panel shows the model trained on CIP that is then used as the pre-trained model to transfer the knowledge to the other three antibiotics. The bottom left panel shows the 5-fold cross-validation scheme. The dataset was firstly split, and 20% was used for testing. The remaining data were used in the cross-validation. The bottom right panel shows our validation scheme for the transfer learning model on an independent public dataset. This figure was created with BioRender.com.

Transfer learning generally uses a known pre-trained model with a large amount of data as the source model [12,14,19,38]. Here, we used the model that performs the best on our AMR dataset as the pre-trained model instead of the public uncorrelated dataset. Thus, we firstly constructed basic CNN architectures for each antibiotic with our data (see Figure 2). The CNN architectures were implemented using the Keras (https://keras.io/, accessed on 15 October 2021) package and TensorFlow (https://tensorflow.org, accessed on 15 October 2021). We evaluated the performance of the CNNs based on accuracy, receiver operating characteristics curve (ROC), and the precision–recall curve (P_R curve), then selected the best-performing model, namely the CIP model, as the source model for transfer learning. The source model based on CIP data not only performed well, but more importantly, the source task was also closely related to the other target tasks, i.e., the prediction of CTX, CTZ, and GEN resistance. We thus transferred the architecture and weights of the source model from the CIP data and retrained the model with CTX, CTZ, and GEN, respectively (see Figure 2). Our dataset was separated into a test set with 20% of the samples, and the remaining data were used for fivefold cross-validation to split the training set and validation set. The public dataset was used as an external validation set to further validate the performance of the models on independent data.

### 2.3. Performance of the Basic CNN Models

**Figure 3:**
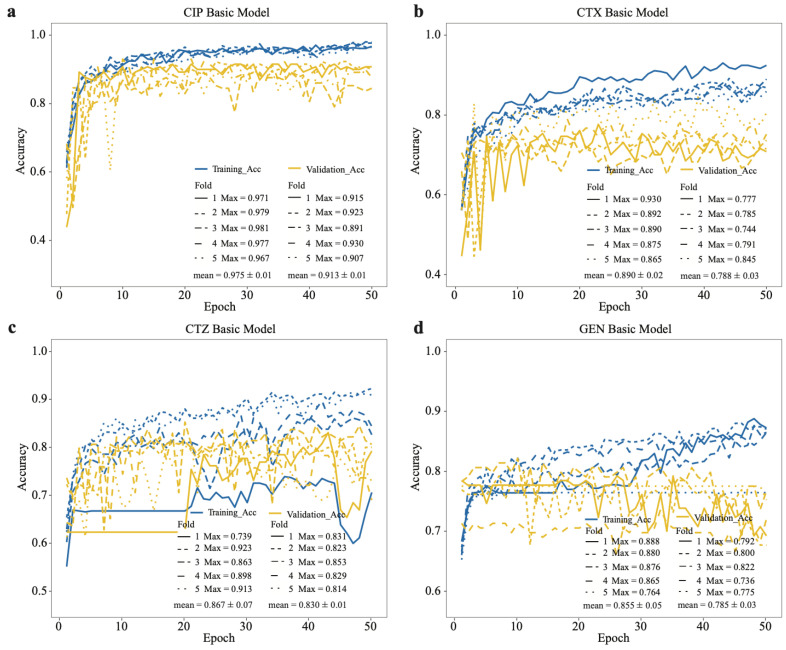
Accuracy of basic CNN models on training and validation datasets based on our dataset. Training accuracy and validation accuracy on (**a**) CIP, (**b**) CTX, (**c**) CTZ, and (**d**) GEN. The legend shows the maximum accuracy in each fold and its mean value.

We built basic convolutional neural network (CNN) models for each antibiotic in our dataset [6]. The dataset was randomly split at 20% to create a testing set, and the remaining data was used in fivefold cross-validation, where we trained the models and fine-tuned the hyper-parameters. We observed that the training accuracy and validation accuracy of the CNN model on CIP data reached a plateau around 0.98 and 0.91, respectively, and there is less bias in each cycle training process (see Figure 3). The training and validation accuracies of the other CNNs trained on the other antibiotics were lower, e.g., the CTX model had accuracies of around 0.89 and 0.79 for training and validation (see Figure 3). For the CTZ data, the training and validation accuracies of the model in fivefold cross-validation were around 0.87 and 0.83. For the GEN data, the accuracies were around 0.86 and 0.79 (see Figure 3). These results indicate that the model on CIP data has the highest accuracy compared with the other models on CTX, CTZ, and GEN data. Thus, we selected the CIP model as the source model for transfer learning.

**Figure 4:**
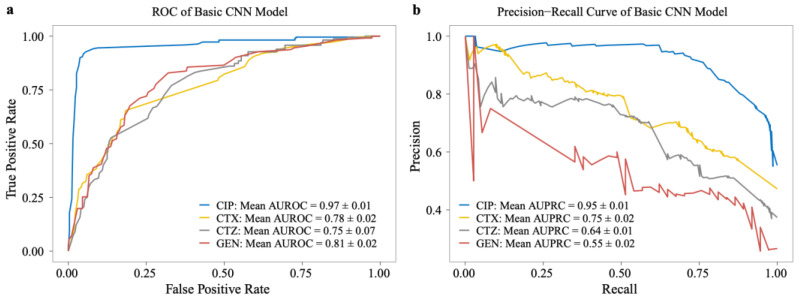
Performance of basic models on the testing dataset of our dataset. (**a**) The ROC curve and (**b**) precision–recall curve (P_R) on CIP, CTX, CTZ, and GEN antibiotics.

We also evaluated the model performance on the testing set using the receiver operating characteristics curve (ROC) and the precision–recall curve (P_R curve). We observed the same results based on the area under the ROC (AUROC) and P_R curves (AUPRC) for CIP (0.97 ± 0.01, 0.95 ± 0.01) and CTX (0.78 ± 0.02, 0.75 ± 0.01) testing data (see Figure 4), which show that the CNN model can generalize well. However, the AUROC and AUPRC are much lower for CTZ (0.75 ± 0.07, 0.64 ± 0.01) and GEN (0.81 ± 0.02, 0.55 ± 0.02) in the testing datasets (see Figure 4).

### 2.4. Deep Transfer Learning Improves the Model Performance on the Minority Group

**Figure 5:**
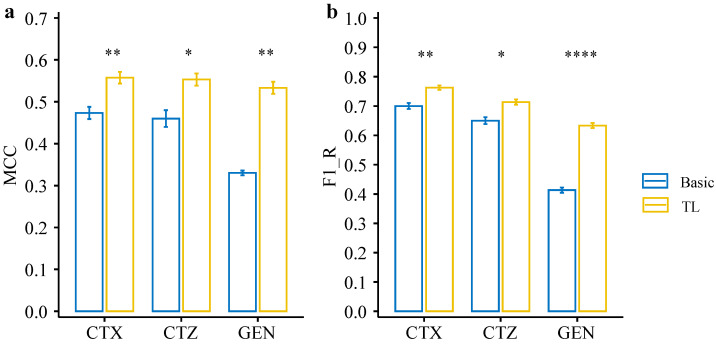
Performance comparison between deep transfer learning models and basic CNN models on the testing set of our dataset. (**a**) MCC of the deep transfer learning models and basic CNN models on each dataset. (**b**) F1_R (F1 resistance) of the deep transfer learning models and basic CNN models on each dataset. Statistical comparisons were performed using the Student’s *t*-test. * *p* < 0.05; ** *p* < 0.01; **** *p* < 0.0001.

**Figure 6:**
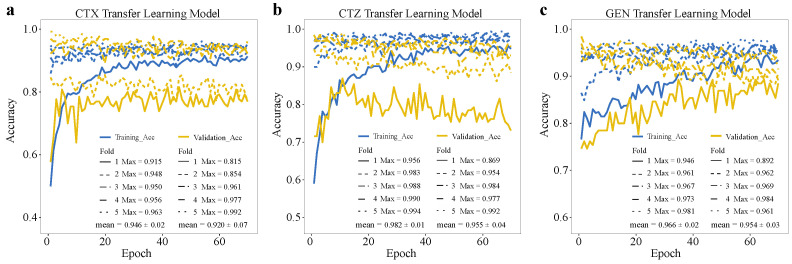
Accuracy of deep transfer learning models on training and validation datasets on our data. Training accuracy and validation accuracy of deep transfer learning models on (**a**) CTX, (**b**) CTZ, and (**c**) GEN. The legends show the maximum accuracy in each fold and its mean value.

Based on the basic CNN model’s performance, we used the model trained on CIP data as the pre-trained model, transferred the learned weights, and retrained the models for CTX, CTZ, and GEN. To evaluate the model performance on the imbalanced datasets, we used the Matthews correlation coefficient (MCC) as one of the evaluation metrics, which is widely used for dealing with binary classification problems on imbalanced data [39,40,41]. Since we are more interested in the resistance phenotype, we also compared the F1 score regarding resistance (F1-R). Our results show that the transfer learning model significantly improves MCC for CTX (*p* = 0.009), CTZ (*p* = 0.023), and GEN (*p* = 0.001) compared with the basic models (see Figure 5a, Table 1). Moreover, the F1-Rs for CTX (*p* = 0.007), CTZ (*p* = 0.014), and GEN (*p* = 6.1 × 10^−5^) of the transfer learning models were significantly higher than the basic models (see Figure 5b, Table 1). We also observed that the maximum accuracy of the transfer learning models stabilize over 0.9 in both the training and validation sets for CTX, CTZ, and GEN. Thus, all of them were significantly improved (Figure 6). These results indicate that transfer learning can improve the model performance, especially for the minority groups, and thus is also applicable for small, imbalanced datasets.

### 2.5. Model Evaluation on Independent Public Data

**Figure 7:**
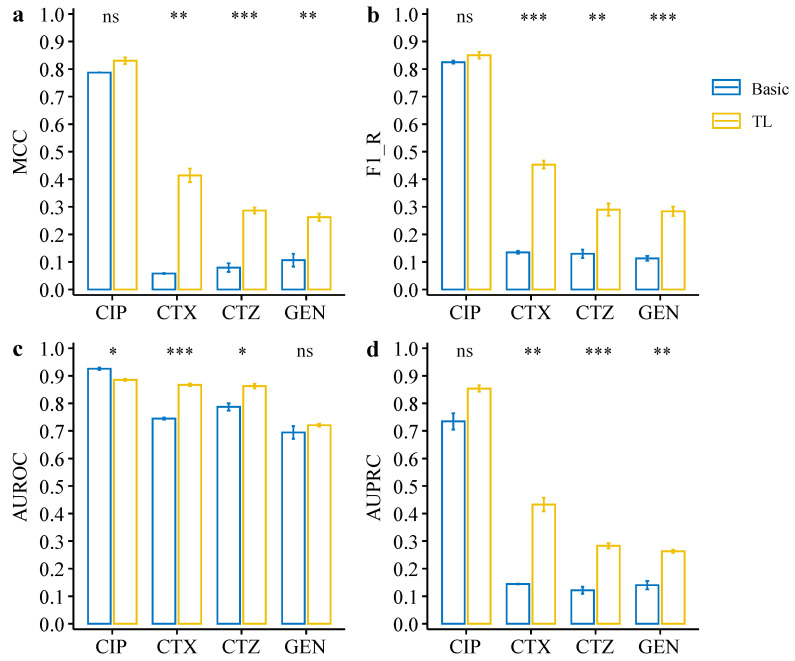
Performance comparison between deep transfer learning models and basic CNN models on the testing dataset of the public dataset. (**a**) MCC of the deep transfer learning models and basic CNN models on each dataset. (**b**) F1_R (F1 resistance) of the deep transfer learning models and basic CNN models on each dataset. (**c**,**d**) AUC of ROC curve (**c**) and precision–recall curve (**d**) of the deep transfer learning models and basic CNN models on each dataset. Statistical comparisons were performed using the Student’s *t*-test. * *p* < 0.05; ** *p* < 0.01; *** *p* < 0.001; ns: not significant.

We further evaluated the deep transfer learning models on an independent public dataset. The public dataset contains data from *E. coli* resistance to the four antibiotics, CIP, CTX, CTZ, and GEN. There is an extreme imbalance between resistant and susceptible phenotypes in this dataset, with a very low number of resistant strains (see Figure 1). We firstly evaluated the model performance based on the MCC metric, which shows that the transfer learning models are significantly better than the original models for CTX (*p* = 4.6 × 10^−3^), CTZ (*p* = 5.6 × 10^−4^), and GEN (*p* = 6.9 × 10^−3^) (see Figure 7a, Table 2). Again, we also observed that the F1-Rs of the transfer learning models were significantly higher than for the basic models for CTX, CTZ, and GEN data (see Figure 7b, Table 2). The MCC and F1-R of the transfer learning model for CIP data were also better than for the basic model. Moreover, we compared the transfer learning models and basic models based on AUROC and AUPRC metrics. The AUROC results suggest that transfer learning significantly improved drug resistance prediction for CTX (*p* = 2.4 × 10^−4^) and CTZ (*p* = 0.012) (see Figure 7c, Table 2). Moreover, the results of AUPRC show that the transfer learning models significantly improved for CTX (*p* = 7.1 × 10^−3^), CTZ (*p* = 4.1 × 10^−4^), and GEN (*p* = 8.1 × 10^−3^) (see Figure 7d, Table 2). Taken together, the results on the public dataset also clearly show that the deep transfer learning models can compensate for class imbalance and thus improve AMR prediction also for small, imbalanced datasets, and thus is also a very promising approach for novel antibiotics in the future where available data on resistance are limited.

## 3. Discussion

In this work, we propose a deep transfer learning model that performs well on small, imbalanced data for AMR prediction. Transfer learning typically pre-trains a model on a larger well-known dataset [30,38]. Here, we used a CNN model on a balanced dataset (CIP dataset) with high accuracy as the pre-trained model. The knowledge obtained from the pre-trained model was then transferred to other datasets with resistance to CTX, CTZ, and GEN. We found that our deep transfer learning model can significantly improve the prediction performance compared with the basic CNN models, ranging from 0.06–0.22 based on different evaluation metrics (see Figure 5, Table 1). Especially, the results indicate that our deep transfer learning model can facilitate the resistance prediction on small, imbalanced datasets. These findings are also supported and validated by an independent evaluation with an unseen, public dataset. The performance was significantly improved, ranging from 0.02–0.35 based on different evaluation metrics (see Figure 7, Table 2). Moreover, we can extend our approach to other species and various antibiotic drugs using our pre-trained model in the future, which will improve the accuracy of resistance prediction and save treatment time, especially for small data sizes with imbalanced labels.

Another interesting result is that we found the performance for CIP data on the public dataset is better than for CTX, CTZ, and GEN public datasets. This result indicates that the closer the correlation between the source task and target task is, the better the performance of the final models. Thus, it is more important to focus on the relevance between the source task and the target tasks when we choose the source domain. The evaluation metrics of the models should be carefully chosen when we are faced with extreme class imbalance. In this article, we provide the commonly used evaluation metrics such as the F1 score, ROC curve, and P_R curve, as well as the evaluation metrics applicable to imbalanced data such as the MCC.

Transfer learning has gained more attention in recent years. For example, Al-Stouhi et al. previously proposed that transfer learning can be used to solve class imbalance problems with inadequate data and provided theoretical and empirical validation on healthcare and text classification applications [10]. Minvielle et al. explored the impact of class imbalance using transfer learning on decision trees [33]. However, only a few studies have been carried out on AMR so far. The proportion of the susceptible and resistant isolates in AMR datasets varies depending on the antibiotic/bacterial species combinations. For the majority of the antibiotics, the AMR data are imbalanced, and the resistant classes of interest are in the minority group. This is particularly true for novel antibiotics in the future, where data of resistant strains are limited. Therefore, our proposed deep transfer learning model paves the way to improve AMR prediction accuracy, as well as for small datasets of novel antibiotics in the future. Moreover, in this analysis, we aimed at identifying secondary mutations that contribute to the resistance directly or indirectly, e.g., compensatory mutations. Thus, we did not include the known resistance genes. Our pre-trained model may not be as effective in predicting resistance due to the transfer of resistance genes compared with resistance due to mutations. Our approach does not need any AMR expert knowledge and can also predict resistance even without knowing the resistance genes by identifying secondary mutations. By combining this data-driven approach with transfer learning, AMR predictions can be significantly improved. It can also be used when only small data are available and information on resistance mechanisms is missing or when the resistance mechanisms are not fully understood yet, e.g., for novel antibiotics.

## 4. Materials and Methods

### 4.1. Data Pre-processing

We performed quality checking and filtering on the raw whole-genome sequencing reads using fastp (v0.23.2) software [42]. The filtered reads were then aligned to the *E. coli* reference genome (*E. coli* K-12 strain. MG1655) using BWA-mem with default parameters [43]. We then called variants from the sequencing data using Bcftools software (v1.14) via the “call” function with default parameters [44]. We extracted SNPs variants, reference alleles, and their positions and merged all isolates based on the positions of reference alleles. We filtered out the loci without variation (N replaces a locus without variation) and retained the existing allele variants of more than half in samples. The final SNP matrix, where each column represents the variant allele, and each row is a sample, was encoded into numerical values by one-hot encoding that can be used for subsequent machine learning. The pre-processing process was carried out according to Ren et al. [6].

### 4.2. Basic CNN Model

**Figure 8:**
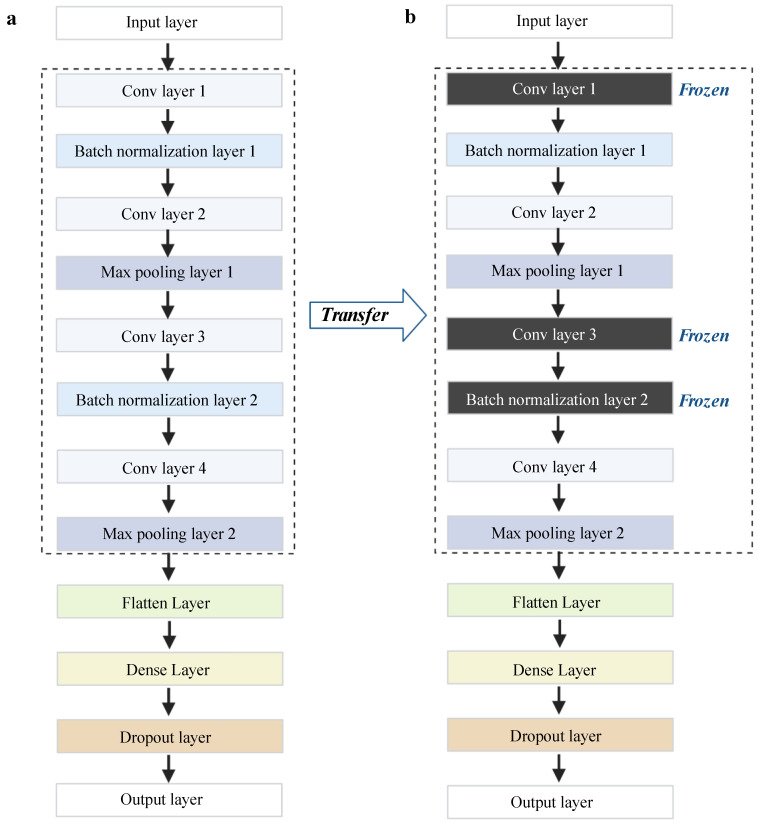
Our framework of basic CNN models and transfer learning models. (**a**) The architecture of the basic CNN models. (**b**) The architecture of the transfer learning models. Conv layer represents convolution layers. This figure was created with BioRender.com.

We used the Keras (https://keras.io/, accessed on 15 October 2021) and Tensorflow (https://tensorflow.org, accessed on 15 October 2021) Python packages to build the CNN models. We evaluated different topologies in the training data and found that a model with 12 layers performed the best. Thus, the architecture of the CNN models (see Figure 8a) contains twelve layers, including four convolutional layers with a kernel size of 3, implemented by the Conv1D function, two pooling layers using the MaxPooling1D function, two batch normalization layers, one flattening layer, one fully connected layer with 128 nodes followed by a dropout layer, and one output layer with the “softmax” activation function. We used the “categorical_crossentropy” loss function and the “Adam” optimizer function to compile the CNN models with 50 epochs. In order to improve the computation speed, we split the data into multiple small batches, with a batch size of 8. 

### 4.3. Deep Transfer Learning Architecture

In order to facilitate the model performance on small, imbalanced data, we employed deep transfer learning. The deep learning architecture is built based on the basic CNN models as previously described (see Figure 8b). In transfer learning, we have to specify the source domain Ds and the target domain Dt and the source task Ts and the target task Tt [38]. Here, we used the CIP dataset from our lab as the source domain Ds; CTX, CTZ, and GEN datasets were used as the target domain Dt. The tasks of Ts and Tt are predicting AMR against different antibiotics. We incorporated two transfer learning strategies, namely fine-tuning and freezing in our work. The fine-tuning strategy is a common deep transfer learning approach based on transferring parameters (weights) from the Ds model to the Dt models [38]. Therefore, we transferred the parameters (weights) of the model trained on CIP into the CTX, CTZ, GEN models, respectively. Furthermore, we froze two normalization layers and one convolution layer and retrained the CNN models on other layers to avoid overfitting [17].

### 4.4. Model Evaluation Metrics

Accuracy, precision, and recall are the basic evaluation metrics for classification models in our study. Accuracy measures the fraction of correct predictions, including positive and negative samples [45]. For binary classification, it can be calculated as follows:

(1) $$\mathrm{Accuracy}\;=\frac{TP+TN}{TP+FP+TN+FN}$$

where *TP* = True Positives (the predicted positive value matches the actual positive value), *TN* = True Negatives (the predicted negative value matches the actual negative value), *FN* = False Negatives (the actual positive value was predicted as negative value), and *FP* = False Positives (the actual negative value was classified as positive value). Precision represents the ratio of true positives to the total predicted positives [45]:

(2) $$\mathrm{Precision}\;=\frac{TP}{TP+FP}$$

Recall refers to how many of the actual positives are captured [45]. It is calculated as follows:

(3) $$\mathrm{Recall}\;=\frac{TP}{TP+FN}$$

F1 score combines precision and recall into one metric [45]:

(4) $$F1=2\times \frac{\mathrm{Precision}\;*\;\mathrm{Recall}}{\mathrm{Precision}+\mathrm{Recall}}$$

The ROC curve (receiver operating characteristic curve) is a chart showing the tradeoff between the true positive rate (TPR) and the false-positive rate (FPR). The PR curve (precision–recall curve) is a graph that combines precision and recall in a single visualization. The higher the area under the curve score, the better the performance of a model. However, accuracy, F1 score, ROC curve, and PR curve are not the best metrics for heavily imbalanced datasets, especially when you are more interested in the minority group. The MCC (Matthews correlation coefficient) is another alternative metric, which is calculated based on the Pearson correlation coefficient between actual and predicted values ranging from [−1, 1] [41]. It is the method of choice for imbalanced datasets [41]:

(5) $$\mathrm{MCC}\;=\frac{TP\times TN-FP\times FN}{\sqrt{\left(TP+FP\right)\times \left(TP+FN\right)\times \left(TN+FP\right)\times \left(TN+FN\right)}}$$

Since some of our datasets are balanced and some are extremely imbalanced, a single metric may not reflect the model performance well. Therefore, we comprehensively evaluated our results based on the above metrics.

## Figures and Tables

**Table 1:** MCC values and F1-R values (F1 on resistance class) of deep transfer learning models and basic CNN models on the testing set of our dataset.

Drugs	CTX		CTZ		GEN	
Metrics	MCC	F1-R	MCC	F1-R	MCC	F1-R
Basic	0.47 ± 0.03	0.70 ± 0.02	0.46 ± 0.03	0.65 ± 0.02	0.33 ± 0.01	0.41 ± 0.02
TL	0.56 ± 0.03	0.76 ± 0.02	0.55 ± 0.03	0.71 ± 0.02	0.53 ± 0.03	0.63 ± 0.02

**Table 2:** MCC values, F1-R values (F1 on resistance class), AUROC, and AUPRC of deep transfer learning models and basic CNN models on the testing set of public dataset.

Drugs	CIP		CTX		CTZ		GEN	
Model	Basic	TL	Basic	TL	Basic	TL	Basic	TL
MCC	0.79 ± 0.00	0.83 ± 0.02	0.06 ± 0.00	0.41 ± 0.04	0.08 ± 0.03	0.29 ± 0.02	0.11 ± 0.04	0.26 + 0.03
F1-R	0.83 ± 0.01	0.85 ± 0.02	0.14 ± 0.01	0.45 ± 0.03	0.13 ± 0.03	0.29 ± 0.05	0.11 ± 0.02	0.28 + 0.04
AUROC	0.93 ± 0.01	0.89 ± 0.01	0.74 ± 0.00	0.87 ± 0.01	0.79 ± 0.02	0.86 ± 0.02	0.69 ± 0.04	0.72 + 0.01
AUPRC	0.73 ± 0.04	0.85 ± 0.02	0.14 ± 0.00	0.43 ± 0.04	0.12 ± 0.02	0.28 ± 0.02	0.14 ± 0.03	0.26 + 0.01

## Data Availability

The datasets from our laboratory used in the current study are publicly available at https://github.com/YunxiaoRen/deep_transfer_learning_AMR (accessed on 15 October 2021). The public dataset information is publicly available at https://doi.org/10.1371/journal.pcbi.1006258.s010 (accessed on 15 October 2021).
